# p97/VCP is highly expressed in the stem-like cells of breast cancer and controls cancer stemness partly through the unfolded protein response

**DOI:** 10.1038/s41419-021-03555-5

**Published:** 2021-03-17

**Authors:** Chuang Li, Yongsheng Huang, Qianqian Fan, Hongyang Quan, Yeqing Dong, Meng Nie, Jiaqi Wang, Fucun Xie, Jiang Ji, Lan Zhou, Zhi Zheng, Lin Wang

**Affiliations:** 1 Department of Physiology, Institute of Basic Medical Sciences, School of Basic Medicine Peking Union Medical College, Chinese Academy of Medical Sciences, 5 Dong Dan San Tiao, Beijing, China; 2 Department of Biochemistry, Institute of Basic Medical Sciences, School of Basic Medicine Peking Union Medical College, Chinese Academy of Medical Sciences, Beijing, China; 3 Peking Union Medical College & Tsinghua University, 5 Dong Dan San Tiao, Beijing, China; 4 Present Address: Division of Nephrology, Department of Medicine, Washington University School of Medicine, 660S. Euclid Ave., St. Louis, MO USA; 5 Present Address: National Research Institute for Family Planning, 12 Da Hui Si, Beijing, China; 6 Present Address: Patent Examination Corporation, State Intellectual Property Office, 2028 Tianfu Avenue South, Chengdu, China; 7 Present Address: School of Pharmacy, Tsinghua University, Beijing, China; 8 Present Address: Department of Physiology, Shanxi Medical University, Taiyuan, China

**Keywords:** Breast cancer, Endoplasmic reticulum, ER-associated degradation

## Abstract

p97/VCP, an evolutionarily concerned ATPase, partakes in multiple cellular proteostatic processes, including the endoplasmic reticulum (ER)-associated protein degradation (ERAD). Elevated expression of p97 is common in many cancers and is often associated with poor survival. Here we report that the levels of p97 positively correlated with the histological grade, tumor size, and lymph node metastasis in breast cancers. We further examined p97 expression in the stem-like cancer cells or cancer stem cells (CSCs), a cell population that purportedly underscores cancer initiation, therapeutic resistance, and recurrence. We found that p97 was consistently at a higher level in the CD44^+^/CD24^−^, ALDH^+^, or PKH26^+^ CSC populations than the respective non-CSC populations in human breast cancer tissues and cancer cell lines and p97 expression also positively correlated with that of SOX2, another CSC marker. To assess the role of p97 in breast cancers, cancer proliferation, mammosphere, and orthotopic growth were analyzed. Similarly as p97 depletion, two pharmacological inhibitors, which targets the ER-associated p97 or globally inhibits p97’s ATPase activity, markedly reduced cancer growth and the CSC population. Importantly, depletion or inhibition of p97 greatly suppressed the proliferation of the ALDH^+^ CSCs and the CSC-enriched mammospheres, while exhibiting much less or insignificant inhibitory effects on the non-CSC cancer cells. Comparable phenotypes produced by blocking ERAD suggest that ER proteostasis is essential for the CSC integrity. Loss of p97 gravely activated the unfolded protein response (UPR) and modulated the expression of multiple stemness and pluripotency regulators, including C/EBPδ, c-MYC, SOX2, and SKP2, which collectively contributed to the demise of CSCs. In summary, p97 controls the breast CSC integrity through multiple targets, many of which directly affect cancer stemness and are induced by UPR activation. Our findings highlight the importance of p97 and ER proteostasis in CSC biology and anticancer therapy.

## Introduction

p97, also known as valosin-containing protein (VCP) and Cdc48, is an evolutionarily conserved ATPase associated with various cellular activities, many of which are related to protein homeostasis or proteostasis^1^. In general, p97/VCP functions as an ATP-dependent segregase, extracting client proteins from macromolecule complexes or membranes and subsequently targeting them for degradation through the ubiquitin–proteasome system^1,2^. Structurally, p97 has two ATPase domains, D1 and D2. ATP hydrolysis in D2 domain provides the energy for the unfolding and dislocation of client proteins.

In mammalian cells, the accumulation of unfolded proteins in the endoplasmic reticulum (ER) activates the unfolded protein response (UPR)^3^. Cancer cells generally encounter a higher degree of unfolded proteins in the ER, i.e., ER stress, due to uncontrolled proliferation, poor vascularization, and hypoxia^4^. Upon ER stress, three ER membrane sensors, IRE1α, PERK, and ATF6α, activate the respective downstream transcription factors, XBP1, ATF4, and ATF6α, which elicit multiple adaptive responses including the ER-associated protein degradation (ERAD)^3^. In the ERAD pathway, p97 functions as a molecular ratchet, pulling the ubiquitinated, unfolded proteins from the ER for proteasomal degradation in the cytosol^5^.

Many types of cancers express high levels of p97, which are often correlated with poor survival, metastasis, and therapeutic resistance^6^. Functionally, a number of inhibitors targeting p97 exhibit potent cytotoxicity in these cancers^6,7^. For example, Eeyarestatin I (Eer I), the first developed p97 inhibitor, has two functional groups. The nitrofuran-containing group in Eer I binds to the D1 domain of p97, whereas the aromatic group localizes Eer I to the ER membrane^8^. Eer I induces the accumulation of polyubiquitinated proteins, inhibits ERAD and activates UPR, as well as inducing apoptosis in hematological cancer cells and synergizing with bortezomib, a proteasome inhibitor^9^. NMS-873 and DBeQ inhibit p97’s ATPase activity and therefore broadly suppress all its functions in the cell^10,11^. NMS-873 and DBeQ also induce cell death in multiple cancers in vitro and in vivo^7,10,11^. Because the inhibition of p97 induces a greater anti-proliferative effect in cancer cells as compared to noncancerous tissues^9,10^, p97 is widely considered as a promising target in cancer therapy. In fact, proteostasis control has emerged as a novel point of intervention in many diseases and especially cancer^12,13^.

Tumor initiating cells, or the stem-like cancer cells, or cancer stem cells (CSCs) are found in many types of cancers including breast cancers. Like normal stem cells, the CSCs express high levels of OCT4 and SOX2, which are pluripotency-determining transcription factors and are frequently overexpressed in poorly differentiated cancers^14^. CSCs are proposed, to a great extent, account for cancer initiation, metastasis, chemotherapy resistance, and relapse. Recently, proteostasis control was shown to be crucial for embryonic and adult stem cells to maintain pluripotency^15^. We previously showed that an appropriate level of UPR activation is required for the integrity of breast CSCs, while excess ER stress ameliorates the CSC population and reduces SOX2 expression^16^. Although p97 is known as essential in cell differentiation and development^17,18^, it is unclear whether p97 plays any role in the integrity and maintenance of CSCs.

In this study, we first established that p97 expression is significantly higher in the CSC populations in human breast cancer tissues and breast cancer cell lines, as compared to the non-CSC populations. Next, we manipulated the activity and expression of p97 using two pharmacological inhibitors, Eer I and NMS-873, which block ERAD and globally inhibit p97, respectively, together with gene silencing and overexpression. The effects on cancer proliferation and the CSC population were investigated using breast cancer cell lines and orthotopic tumor models. Then we characterized the mechanisms underlying p97’s action on breast CSCs. We showed that, similarly as p97 depletion, Eer I and NMS-873 comparably suppressed cancer growth and the CSC population. p97 depletion or inhibition markedly suppressed the proliferation of the ALDH^+^ CSCs or CSC-enriched mammospheres, while exhibiting much less or insignificant inhibitory effects in the non-CSC cancer cells. Loss of p97 gravely activates the UPR and modulates the expression of multiple stemness and pluripotency regulators, including C/EBPδ, c-MYC, SOX2, and SKP2, which collectively contribute to CSC demise.

## Materials and methods

### Cell culture and reagents

Human breast cancer cell lines MCF-7 and MDA-MB-231 and human immortalized mammary epithelial cell line MCF10A were gifts from Drs. Yunping Luo and Ye Zhang (Chinese Academy of Medical Sciences, Peking Union Medical College). These cell lines were authenticated using short tandem repeat DNA profiling by Microread Genetics (Beijing, China) and cultured as previously described^16^. Eer I and NMS-873 were purchased from Calbiochem. Other chemicals were from Sigma-Aldrich unless stated otherwise.

### Immunohistochemistry

Immunohistochemical staining was performed using the SPlink Detection Kit (Zsbio, Beijing, China) as previously reported^16^. The primary antibodies used in this study include: p97 (a generous gift from Dr. Hartmann Peterson, University of Copenhagen), PCNA (Cell Signaling Technology, 2586), cleaved CASPASE-3 (Cell Signaling Technology, 9664), CD31 (Santa Cruz, sc-46694), CD44 (Thermo Scientific, 156-3C11), CD24 (Thermo Scientific, SN3b), OCT4 (Abcam, ab18976), c-MYC (Cell Signaling Technology, 5605), SKP2 (Proteintech, 15010-1-AP), and SOX2 (Proteintech, 11064-1-AP). Diaminobenzidine was used to visualize the immunolabeled proteins. Finally, the sections were counterstained with hematoxylin and eosin, examined and photographed with an Olympus bright-field microscope.

### Tissue microarray

Tissue microarrays consisting of human breast cancer tissues and normal breast tissue were purchased from Alenabio Inc (Xi’an, China) and Zhuoli Biotech (Shanghai, China), which acquired informed consent from the participating subjects. Immunohistochemical staining of p97, CD44, CD24, SOX2, c-MYC, and SKP2 were performed using the antibodies described above. The staining intensity was scored on a scale of 0 to 3: 0 (negative or very weak), 1 (weak), 2 (moderate), and 3 (strong). Score <1 was considered as negative, and score ≥1 as positive. Expression levels in each sample were calculated by summating the percentage of area stained at each intensity multiplied by the scored intensity (e.g., 1, 2, or 3). Chi-squared tests were used to determine any statistically significant correlation between p97 level and CD44^+^/CD24^−^ population or the levels of SOX2, c-MYC, and SKP2, as well as the correlation between p97 levels and the pathological grade and TNM staging. As a tissue microarray of too small size lacks statistical power to detect associations of protein expression with outcome, we used multiple tissue microarrays totaling 291 patient samples to analyze the correlation of p97 expression with the pathological grade or TNM staging. Similarly, we used tissue microarrays of 75–100 samples to examine the correlation between p97 expression and CD44^+^/CD24^−^ CSC population or the expression of other stemness regulators.

### RNA extraction and real-time quantitative PCR (qPCR)

Total RNAs were extracted using TRIZOL reagent (Invitrogen). Real-time qPCR was performed using the StepOnePlus Real-time PCR system (Applied Biosystems) with SYBR Green PCR Master Mix (Transgen, Beijing, China) as previously described^16^. The primers were designed using Primer-BLAST (National Center for Biotechnology Information, NCBI) with sequence as follows: *p97*: forward, 5′-AAACCGTGGTAGAGGTGCCA-3′; reverse, 5′-CTTGGAAGGTGTCATGCCAA-3′; *SOX2*: forward, 5′-AACCAGCGCATGGACAGTTA-3′; reverse, 5′-CGAGCTGGTCATGGAGTTGT-3′; *OCT4*: forward, 5′-TGGAGAAGGAGAAGCTGGAGCAAAA-3′; reverse, 5′-GGCAGATGGTCGTTTGGCTGAATA-3′; *BIP*: forward, 5′-GAACGTCTGATTGGCGATGC-3′; reverse, 5′-ACCACCTTGAACGGCAAGAA-3′; *CHOP*: forward, 5’- AGCCAAAATCAGAGCTGGAA-3′; reverse, 5′-TGGATCAGTCTGGAAAAGCA-3′; *HIF1A*: forward, 5’-GATGTAATGCTCCCCTCACCCAAC-3′, reverse, 5′-CACTGGGACTATTAGGCTCAGGTG-3′; *CEBPD*: forward, 5′-AGTTCTTGGGACATAGGAGCGCA-3′; reverse, 5′- GTACCTTAGCTGCATCAACAGGAG-3′; *SKP2*: forward, 5′-GGCTGAAGAGCAAAGGGAGT-3′; reverse, 5′-GGGAGGCACAGACAGGAAAA-3′; *GAPDH*: forward, 5′- AGCCACATCGCTCAGACAC-3′; reverse, 5′-GCCCAATACGACCAAATCC-3′. The results represent the average of three independent experiments.

### Immunoblotting

Total cellular lysates were prepared and analyzed by SDS-polyacrylamide gel electrophoresis, followed by immunoblotting using a standard protocol^16^. The primary antibodies used in this study include: p97 (a generous gift from Dr. Hartmann Peterson, University of Copenhagen), ubiquitin (Santa Cruz, sc-8017), OCT4 (Proteintech, 11263-1-AP), SOX2 (Proteintech, 11064-1-AP), PERK (Cell Signaling Technology, 5683), phospho-PERK (Santa Cruz, sc-32577), ATF6α (Abcam, ab122897), BIP (Santa Cruz, sc-1051), XBP1 (BioLegend, 619502), ATF4 (Cell Signaling Technology, 11815), HIF-1α (Proteintech, 20960-1-AP), C/EBPδ (Santa Cruz, sc-636), c-MYC (Cell Signaling Technology, 5605), SKP2 (Proteintech, 11064-1-AP), β-actin (Molecular Biological Laboratories, PM053), and GAPDH (Cell Signaling Technology, 5174).

### Flow cytometry and fluorescence-activated cell sorting (FACS)

The assessment of CD44^+^/CD24^−^ CSC population and the isolation of CSCs and non-CSC cells based on three independent criteria (CD44^+^/CD24^+/high^ and CD44^+^/CD24^−/low^ cells from MCF-7 cells using FITC- and PE-conjugated anti-CD44 and anti-CD24 antibodies; ALDH^+/high^ and ALDH^−/low^ cells from MDA-MB-231 cells using ALDEFLOUR assay; PKH26^+^ and PKH26^−^ cells from MDA-MB-231 cells using a PKH26 labeling kit) were carried out as previously reported^16^. In these separations, the CD44^+^/CD24^−/low^, ALDH^+/high^, and PKH26^+^ populations were considered to represent the breast CSCs.

### Proliferation, apoptosis, invasion, and mammossphere assays

In vitro cell viability/proliferation, apoptosis, invasion, and mammosphere formation and maintenance were performed as previously described^16^.

### Xenograft tumor model

Five BALB/c nude mice (Vital River Laboratories, Beijing, China) per group were used in the xenograft tumor growth experiments. After acclimatization, mice were randomly selected for treatment with inhibitors or dimethyl sulfoxide (DMSO) as a vehicle control. All mouse experiment protocols were approved by the Institutional Animal Care and Use Committee of Peking Union Medical College and Chinese Academy of Medical Sciences. All animal care and experimental methods were carried out following the ARRIVE guidelines for animal experiments. No blinding was involved in animal studies.

For nude mice experiments not involving CSCs, 5 × 10^6^ MDA-MB-231 cells were injected into the breast fat pads of 4-week-old female BALB/c nude mice. For inhibitor treatment, Eer I and NMS-873 (5 mg/kg mouse bodyweight) was directly injected into the tumor at the day 11, 17, 23, and 29 after xenograft transplant. Intratumoral injection instead of intraperitoneal or tail vein injection was chosen to minimize the systemic effect of p97 inhibitors on nude mice. Tumor was measured every week and the tumor volume was determined as previously described^16^. After 7 weeks, mice were sacrificed and tumor tissues were harvested for immunohistochemistry and flow cytometry analysis.

For CSC nude mice experiment, ALDH^+/high^ and ALDH^−/low^ cells were separated from MDA-MB-231 cells by FACS. Then 1 × 10^5^ ALDH^+/high^ and ALDH^−/low^ cells were subcutaneously injected. p97 inhibitors were injected at the day 21, 28, 35, 42, 49 after xenograft was implanted. Tumor was measured every week. After 8 weeks, mice were sacrificed and tumors were resected and weighed.

### Adenovirus infection

Recombinant adenovirus expressing *p97* was generously provided by Dr. Wei Li (Institute of Zoology, Chinese Academy of Sciences). The HEK-293 cells were used for adenovirus amplification. For infection, viral concentrations were at 0.2–1 × 10^6^ plaques per ml.

### RNA interference

MDA-MB-231 cells were seeded in 6-well plates and transfected with siRNA oligonucleotides (50 nmol per well) with RNAiMAX (Invitrogen). Seventy-two hours after transfection, cells were harvested for further analysis. The siRNAs were synthesized by GenePhama (Shanghai, China) as follows: *p97*: siRNA1, 5′-GAAUAGAGUUGUUCGGAAU-3′; siRNA2, 5′-GGAGGUAGAUAUUGGAAUU-3′, and *SKP2*: 5′-GCAGACCTTAGACCTCACAGGTAAA-3′. *CEBPD* siRNAs were purchased from Dharmacon: D-010453-01 (5′- GGGAGAAGAGCGCCGGCAA-3) and D-010453-02 (5′-GAGAAGAGCGCCGGCAAGA-3′). Non-targeting (NT) siRNA: 5′-UUCUCCGAACGUGUCACGU-3′ was purchased from GenePharma (Shanghai, China). A recombinant lentivirus encoding a doxycycline-inducible shRNA construct targeting *p97* was designed using a 21-mer sequence (AACAGCCATTCTCAAACAGAA) as previously reported^19^ and synthesized by GeneChem Inc. (Shanghai, China).

### Microarray analysis

Total RNAs were isolated from Eer I- and NMS-873-treated MDA-MB-231 cells and analyzed by Human OneArray Plus Microarray (Zhuoli Biotech, Shanghai, China). The differentially expressed genes (DEGs) were identified as change >2 fold and *p* < 0.05. Gene ontology (http://www.geneontology.org) and KEGG Mapper (http://www.genome.jp/kegg/pathway.html) were used to analyze the molecular functions, biological processes, and canonical pathways that DEGs are involved. The gene expression profiles can be downloaded from https://figshare.com/s/86c3fcce65a7718f94f3.

### Statistical analysis

Data were analyzed using GraphPad Prism5 (San Diego, CA). Significant difference was determined using the two-tailed student’s *t* test or the Pearson’s chi-squared (*χ*^2^) test, wherever appropriate.

## Results

### p97 expression is elevated in the breast CSC population

**Fig. 1:**
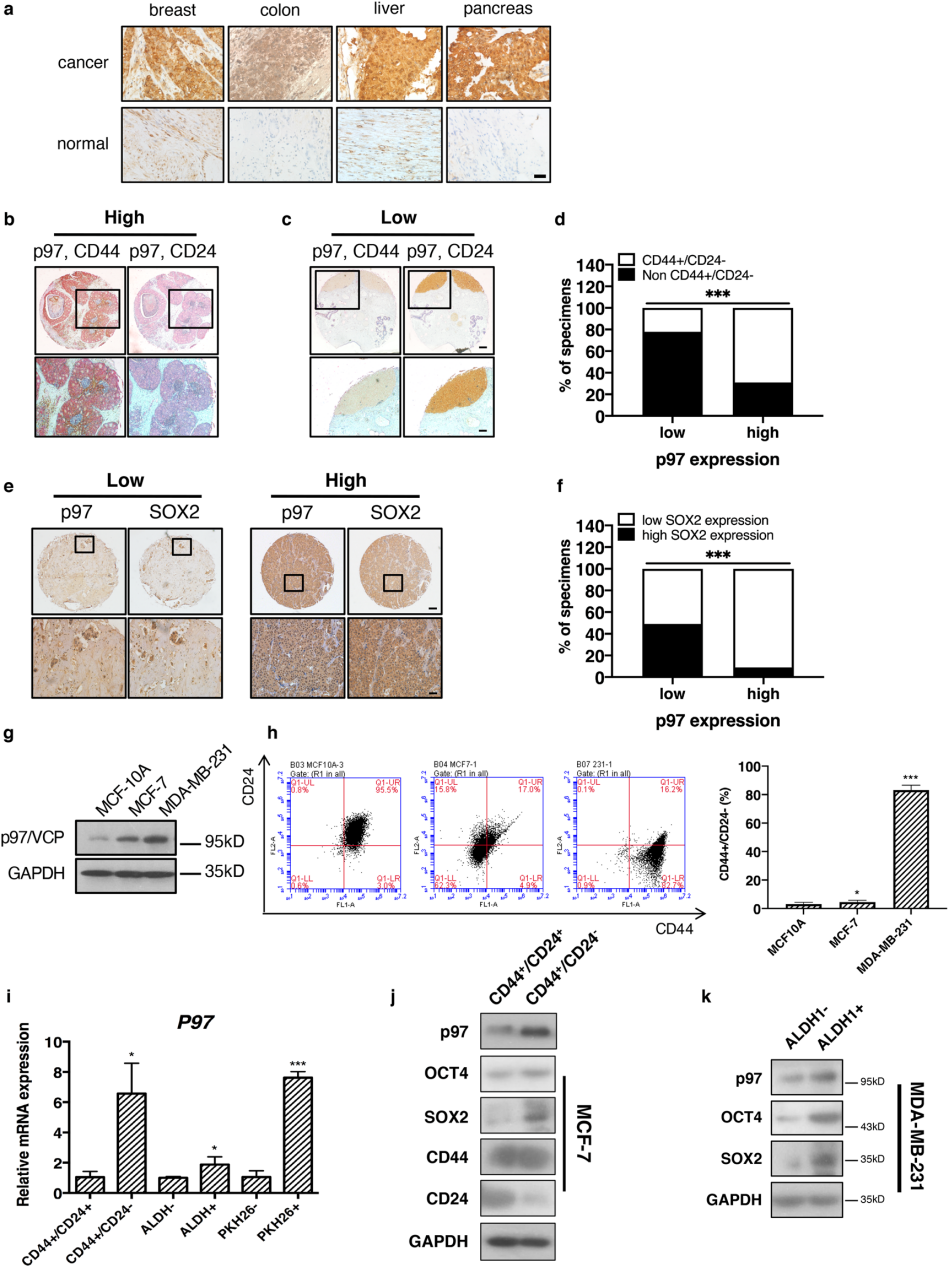
p97 expression is higher in the breast CSC population. **a** Representative immunohistochemical staining of p97 in human breast, colon, liver, and pancreatic cancers and adjacent noncancerous tissues. Bar: 20 μm. **b** Representative immunohistochemical staining of high-level p97 (red) co-stained with CD44 (brown, left) and CD24 (brown, right) in two consecutive serial sections of human breast cancer tissues. **c** Representative immunohistochemical staining of low-level p97 (red) co-stained with CD44 (brown, left) and CD24 (brown, right) in two consecutive serial sections of human breast cancer tissues. Bar: upper 200 μm, lower 50 μm. Note that p97 primarily localized to CD44^+^/CD24^−^ cells but not other cells. **d** Percentages of p97 expression in CD44^+^/CD24^−^ cells and non-CD44^+^/CD24^−^ (CD44^−^/CD24^−^, CD44^−^/CD24^+^, and CD44^+^/CD24^+^) cells in breast cancer tissues (*n* = 75). **e** Immunohistochemical staining of SOX2 and p97 in the consecutive serial sections of breast cancer tissues. SOX2 and p97 expressions were classified as low and high. Bar: upper 200 μm, lower 50 μm. **f** Correlation analyses of SOX2 and p97 expression in breast cancer tissues (*n* = 75). **g** Immunoblotting of p97 in lysates of MCF10A, MCF-7, and MDA-MB-231 cells. GAPDH was used as a loading control. **h** Left: flow cytometry analysis of CD44 and CD24 in MCF10A, MCF-7, and MDA-MB-231 cells. Right: the CD44^+^/CD24^−^ percentages. **i** qPCR analysis of *P97* mRNA in CD44^+^/CD24^−^, non-CD44^+^/CD24^−^, ALDH^+^, ALDH^−^, PKH26^+^, and PKH26^−^ populations. **j**, **k** Immunoblotting analysis of p97, OCT4, and SOX2 in CD44^+^/CD24^−^, CD44^−^/CD24^−^, ALDH^+^, and ALDH^−^ populations isolated from MCF-7 and MDA-MB-231 cells. GAPDH was used as a loading control. Data were shown as mean + SD. *P < 0.05 and ***P < 0.001.

First, immunohistochemistry showed that p97 level was higher in human breast cancer tissues, compared with the surrounding noncancerous tissues (Fig. 1a). Similar trend was seen in colon, liver, and pancreatic cancers (Fig. 1a). Next, we found that p97 expression was positively correlated with the histological grade, tumor size, and lymph node metastasis (significant for all three categories) in collectively 291 cases of breast cancers (Table 1). Under the same condition, we previously found that the levels of PERK and ATF6α (and IRE1α to a lesser extent) also positively correlate with the grades and TNM stages of breast cancers^16^.

**Table 1:** Correlation between p97 expression and clinicopathological features of breast cancer patients (*n* = 291).

	p97 clinicopathological features in breast cancer			
		Low (142)	High (149)	*p* value
Sex	Female	142	149	N/A
	Male	0	0	
Age (years)	>55	27	38	0.184
	<55	115	111	
Grade	Benign	25	0	<0.001
	I	7	3	
	II	94	106	
	III	16	40	
Tumor size	T0	25	0	<0.001
	T1	11	6	
	T2	81	107	
	T3–T4	25	36	
LN metastasis	No	110	98	0.027
	Yes	32	51	

*p* values were calculated using the chi-squared test.

*LN* lymph node, *N/A* non applicable.

CD44^+^/CD24^−^ population is considered as the tumorigenic (tumor initiating) cells or CSCs in breast cancer^20^. CD44^+^/CD24^−/low^ population has also been used to predict the prognosis of basal-like breast carcinomas^21^. Double immunohistochemistry of human breast cancer tissues showed that p97 expression was significantly higher (*p* = 3.827 × 10^−6^) in CD44^+^/CD24^−^ cells than in the non-CD44^+^/CD24^−^ cells (i.e., CD44^−^/CD24^−^, CD44^−^/CD24^+^, and CD44^+^/CD24^+^ cells) (Fig. 1b–d and Table S1). 68.7% of CD44^+^/CD24^−^ cells expressed high levels of p97, whereas 78.2% of the non-CD44^+^/CD24^−^ cells expressed low or undetectable levels of p97 (Table S1). We also found a positive correlation between the levels of p97 and SOX2, a pluripotency regulator associated with normal stem cells and CSCs (Fig. 1e, f and Table S2). These data demonstrate that p97 expression is higher in the CSC population from breast cancer patients.

Next, we examined p97 expression in three mammary cell lines with progressive malignancy: the immortalized mammary epithelial MCF10A cells, luminal adenocarcinoma MCF-7 cells, and poorly differentiated, triple (estrogen receptor, progesterone receptor, and HER2) negative carcinoma MDA-MB-231 cells. p97 expression was higher in MDA-MB-231 cells, as compared to MCF-7 cells and MCF10A cells (Fig. 1g). These results suggest that higher levels of p97 either contribute to or are necessary for breast cancer malignancy. Furthermore, CD44^+^/CD24^−/low^ population steadily increased from MCF10A and MCF-7 to MDA-MB-231 cells (Fig. 1h).

We then analyzed p97 expression in the CSCs and non-CSC cells isolated from MCF-7 and MDA-MB-231 cells using three independent CSC criteria^16^. CD44^+^/CD24^−/low^ and aldehyde dehydrogenase (ALDH) positive cells have been proposed to represent the mesenchymal, basal and epithelial, luminal types of breast CSCs, respectively^22^, while PKH26^+^ cells isolated from breast cancer-derived spheres are quiescent and CSC-like^23^. qPCR analysis showed that *p97* mRNA levels were consistently higher in CD44^+^/CD24^−^ cells versus non-CD44^+^/CD24^−^ cells, in ALDH^+^ cells versus ALDH^−^ cells, and in PKH26^+^ cells versus PKH26^−^ cells (Fig. 1i). Immunoblotting confirmed elevated expression of p97 in CD44^+^/CD24^−^ population versus CD44^+^/CD24^+^ population and in ALDH^+^ cells versus ALDH^−^ cells (Fig. 1j, k). The expression of SOX2 and OCT4 was also higher in the breast CSCs, as compared with the non-CSC cancer cells (Fig. 1j, k). We previously also reported increased expression of UPR factors (IRE1α, PERK, ATF6α, and ATF4) in the breast CSCs^16^.

### Inhibition of p97 reduces breast cancer growth and the CSC population

**Fig. 2:**
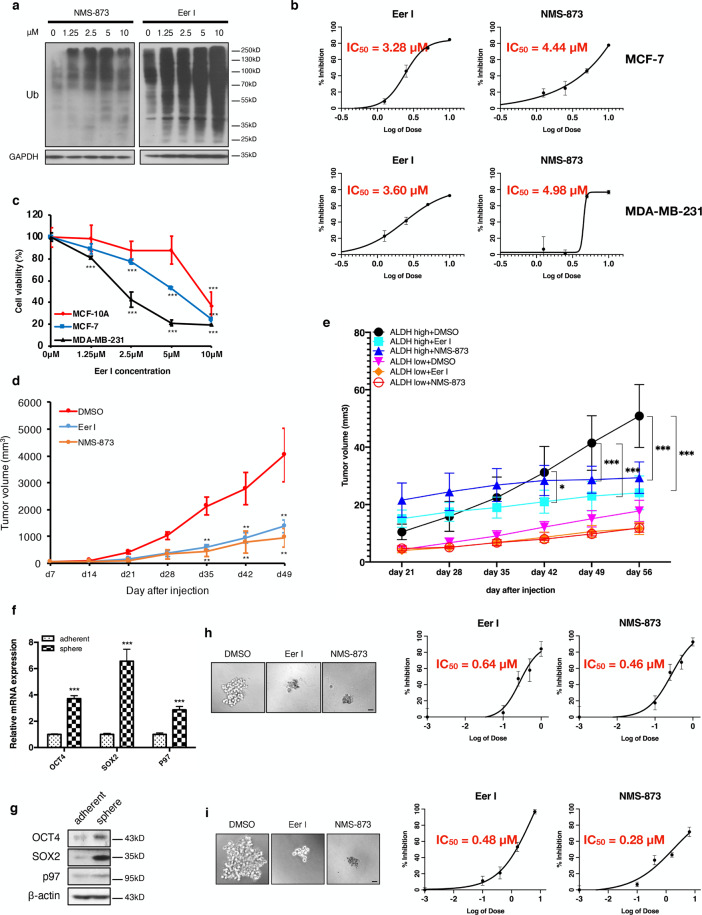
p97 inhibition reduces breast cancer growth, the CSC population, and mammosphere formation. **a** MDA-MB-231 cells were treated with 1.25, 2.5, 5, and 10 μM Eer I and NMS-873 for 24 h. DMSO was used as a vehicle control. The ubiquitinated proteins in cell lysates were analyzed by immunoblotting. GAPDH was used as a loading control. **b** Proliferation of MCF-7 and MDA-MB-231 cells treated with Eer I and NMS-873, with IC50 values indicated. **c** MCF10A, MCF-7, and MDA-MB-231 cells were treated with 1.25, 2.5, 5, and 10 μM Eer I for 24 h and cell viability was measured. **d** Growth of orthotopic tumors formed by MDA-MB-231 cells and treated with p97 inhibitors or DMSO. **e** Growth of orthotopic tumors formed by ALDH^+^ CSCs and ALDH^−^ non-CSC cells and treated with p97 inhibitors or DMSO. **f**, **g** qPCR and immunoblotting analysis of p97, OCT4, and SOX2 in adherent and spheroid MDA-MB-231 cells. β-actin serves as a loading control for immunoblotting. **h** Twenty-four hours after MDA-MB-231 cells were seeded, Eer I and NMS-873 were added to the culture at 0.1, 0.4, 1.6, and 6.4 μM for 7 days to assess their impact on mammosphere formation. DMSO was used as a vehicle control. Left: representative images of the spheres treated with 6.4 μM Eer I or 6.4 μM NMS-873. Bar: 100 μm. Right: the percentage of spheres formed under each condition relative to the control group, with IC50 values indicated. **i** Eer I and NMS-873 were added to the culture at 0.1, 0.25, 0.5, and 1 μM for 7 days after the secondary spheres were formed to assess their impact on mammosphere maintenance. Left: representative images of the spheres treated with 1 μM Eer I or 1 μM NMS-873. Bar: 100 μm. Right: the percentage of spheres formed under each condition relative to the control group, with IC50 values indicated. Data were shown as mean + or ± SD. *P < 0.05, **P < 0.01, and ***P < 0.001.

Because the level and activity of p97 critically affect growth in many cancers, we used two specific inhibitors, Eer I and NMS-873, to treat MDA-MB-231 and MCF-7 cells. Consistent with p97’s central role in ubiquitin-mediated degradation, treatment with Eer I and NMS-873 resulted in the accumulation of polyubiquitinated proteins in MDA-MB-231 cells (Fig. 2a). Eer I and NMS-873 treatment markedly reduced the proliferation of MDA-MB-231 and MCF-7 cells in a dose-dependent manner (Fig. 2b). Similar inhibitory effects were obtained with hepatocarcinoma HepG2, colon carcinoma HCT-116, and pancreatic carcinoma PANC-1 cells (Fig. S1). Notably, the growth inhibitory effect of Eer I was more pronounced in MDA-MB-231 and MCF-7 cells, compared with MCF10A cells, which are not cancerous cells and express much less p97 (Figs. 1g and 2c). This result is consistent with the notion that p97 inhibition induces a greater anti-proliferative effect in cancer cells as compared to normal tissues. As reported previously, Eer I treatment also induced apoptosis in MDA-MB-231, MCF-7, HepG2, HCT-116, and PANC-1 cells, as well as reducing the CD44^+^/CD24^−^ population in MDA-MB-231 cells in vitro^16^. In addition, Eer I treatment reduced the invasion of MDA-MB-231 cells (Fig. S2a).

In the orthotopic tumor model, treatment with Eer I and NMS-873 significantly reduced the growth of MDA-MB-231 cells in athymic mice (Fig. 2d). As shown by immunohistochemistry, p97 inhibition reduced cancer cell proliferation (PCNA) and intratumoral angiogenesis (CD31), as well as inducing apoptosis (cleaved CASPASE-3) (Fig. S2b). Under these conditions, the expression of CD44 and OCT4 was reduced in the Eer I-treated tumors, while CD24 levels increased (Fig. S2b). Flow cytometry analysis further verified the decrease in CD44^+^/CD24^−^ CSC population in the Eer I-treated tumors (Fig. S2c). To further characterize the importance of p97 in CSC-mediated tumorigenesis in vivo, we isolated ALDH^+^ CSCs and ALDH^−^ non-CSC cells from MDA-MB-231 cells and inoculated equal number of ALDH^+^ and ALDH^−^ cells into nude mice. The separation of CSCs and non-CSC cells from MDA-MB-231 cells using CD44^+^/CD24^−^ criteria is less attainable, as over 80% MDA-MB-231 cells are CD44^+^/CD24^−^.

The ALDH^+^ CSCs grew more rapidly and formed larger tumors in nude mice, compared with ALDH^−^ non-CSC cells (Fig. S3a). This is analogous to the greater tumorigenicity of the mammospheres derived from MDA-MB-231 cells, as compared to the adherent MDA-MB-231 cells^16^. Eer I and NMS-873 treatment led to a significant reduction in the volume (from day 42) and weight (at the time of sacrifice, day 56) of the tumors formed by ALDH^+^ CSCs, while the growth reduction was much smaller and insignificant in tumors formed by ALDH^−^ non-CSC cells (Figs. 2e and S3b). Together, these data show that: (1) the loss of p97 activity impairs cancer growth and the CSC population of MDA-MB-231 cells both in vitro and in vivo; (2) blocking ERAD by Eer I produces almost identical phenotypes as global inhibition of p97’s ATPase activity by NMS-873; (3) the breast CSCs are much more sensitive to the inhibition of p97 than non-CSC cells.

### p97 is required for the integrity of the CSC-enriched mammospheres

Mammosphere cultures enrich the CSC population with greater tumorigenicity and favor the selection of mesenchymal CSCs^24,25^. At both the mRNA and protein levels, p97 expression was significantly higher in mammospheres than the adherent MDA-MB-231 cells (Fig. 2f, g). The expression of *OCT4* and *SOX2* was also higher in mammospheres, consistent with CSC enrichment (Fig. 2f, g). Functionally, Eer I and NMS-873 treatment reduced both the formation and maintenance of the mammospheres in a dose-dependent fashion (Fig. 2h, i). Based on IC50s values, the CSC**-**enriched mammospheres were more sensitive to loss of p97 activity than adherent MDA-MB-231 cells (Fig. 2h, i, compared with 2b). These findings indicate that CSC-enriched mammospheres can serve as another amendable system, in which the functional impact of p97 and ERAD inhibition can be easily investigated.

### Validation of p97 inhibition phenotypes by gene overexpression and silencing

**Fig. 3:**
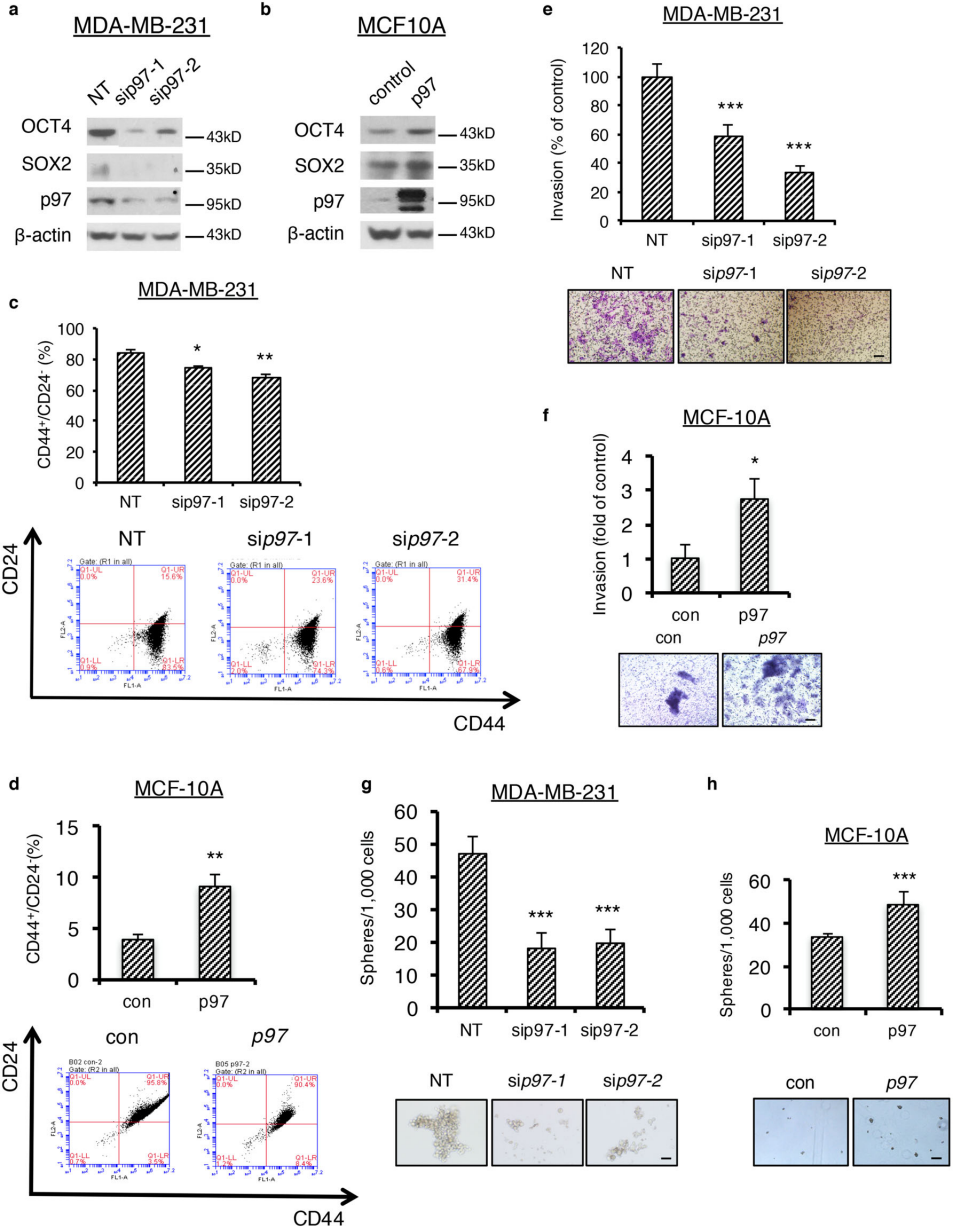
p97 levels control stemness regulator expression, CSC population, cancer invasiveness, and mammosphere formation. **a**, **b** Immunoblotting analysis of OCT4, SOX2, and p97 in MDA-MB-231 cells with *p97* silenced or MCF10A cells overexpressing p97. β-actin serves as a loading control. **c**, **d** Flow cytometry analysis of CD44^+^/CD24^−^ population in MDA-MB-231 cells with *p97* silenced or MCF10A cells overexpressing p97. Upper: the percentage of CD44^+^/CD24^−^ cells. Lower: flow cytometry analyses. **e**, **f** Transwell invasion of MDA-MB-231 cells with *p97* silenced or MCF10A cells overexpressing p97. Upper: percentage of the invaded cancer cells relative to the control group. Lower: representative images of crystal purple staining. Bar: 100 μm. **g**, **h** Mammosphere formation of MDA-MB-231 cells with *p97* silenced or MCF10A cells overexpressing p97. Upper: the percentage of spheres formed under each condition relative to the control group. Lower: representative images of the spheres. Bar: 100 μm. Data were shown as mean + SD. *P < 0.05, **P < 0.01, and ***P < 0.001.

Next, we validated our findings from pharmacological inhibitors using *p97* overexpression and silencing in MCF10A and MDA-MB-231 cells, which express low and high levels of p97, respectively (Fig. 3a). *P97* knockdown in MDA-MB-231 cells greatly reduced the levels of OCT4 and SOX2, the CD44^+^/CD24^−^ CSC population, the Matrigel invasion, and the mammosphere formation (Fig. 3a, c, e, g). Conversely, *p97* overexpression in MCF10A cells produced the opposite phenotypes: increased levels of OCT4 and SOX2, increased CD44^+^/CD24^−^ CSC population, increased Matrigel invasion, and increased mammosphere formation (Fig. 3b, d, f, h). To confirm the p97 knockdown phenotype achieved with siRNA transient transfection, MDA-MB-231 cells were transfected with a doxycycline (dox)-inducible construct encoding *p97* shRNA (Fig. S4a, b). Marked reduction in mammospheres was concomitant with the reduced expression of p97. These results verified that p97 expression, just as its activity, is critical for breast CSC formation, cancer pluripotency, and invasiveness.

### Loss of p97 reduces the expression of MYC and SKP2

**Fig. 4:**
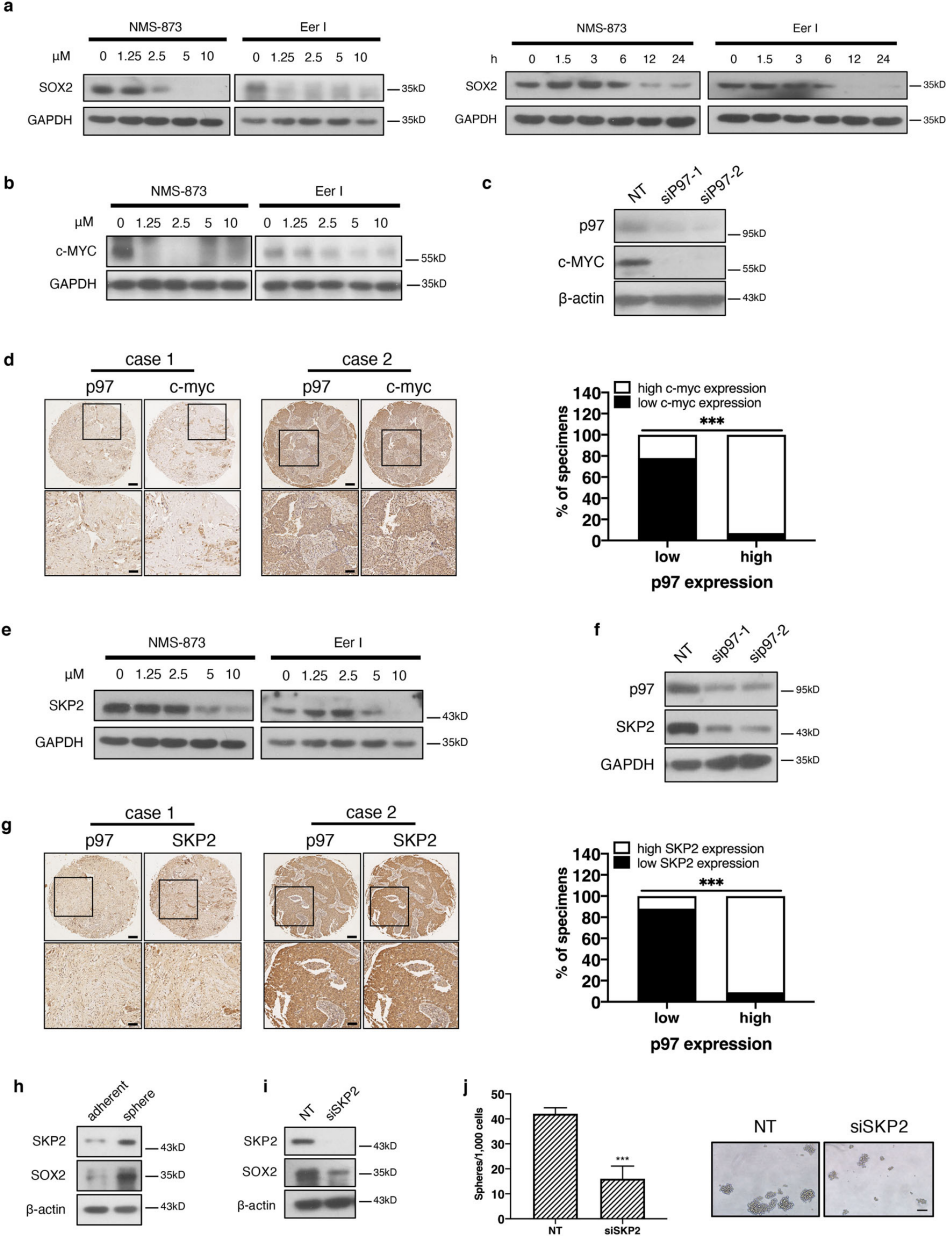
Loss of p97 downregulates c-MYC and SKP2. **a** Immunoblotting analysis of SOX2 from the MDA-MB-231 cells treated with Eer I or NMS-873. GAPDH serves as a loading control. **b** Immunoblotting analysis of c-MYC from Eer I and NMS-873 treated MDA-MB-231 cells. GAPDH serves as a loading control. **c** Immunoblotting analysis of c-MYC from the MDA-MB-231 cells with *p97* silenced. β-actin serves as a loading control. **d** c-MYC expression correlates with that of p97 in human breast cancer tissues. Left: representative immunohistochemical staining of c-MYC and p97 in the consecutive serial sections of breast cancer tissues. c-MYC and p97 expressions were classified as low and high. Bar: upper 200 μm, lower 50 μm. Right: correlation analyses of c-MYC and SOX2 expression in breast cancer tissues (*n* = 98). **e**, **f** Immunoblotting analysis of SKP2 from Eer I and NMS-873 treated MDA-MB-231 cells or MDA-MB-231 cells with *p97* silenced. GAPDH serves as a loading control. **g** SKP2 expression correlates with that of p97 in human breast cancer tissues. Left: representative immunohistochemical staining of SKP2 and p97 in the consecutive serial sections of breast cancer tissues. SKP2 and p97 expressions were classified as low or high. Bar: upper 200 μm, lower 50 μm. Right: correlation analyses of SKP2 and p97 expression in breast cancer tissues (*n* = 75). **h** Immunoblotting analysis of SOX2 and SKP2 from mammospheres and adherent MDA-MB-231 cells. β-actin serves as a loading control. **i** Immunoblotting analysis of SOX2 and SKP2 from mammospheres with *SKP2* silenced. β-actin serves as a loading control. **j** Mammosphere formation of MDA-MB-231 cells with *SKP2* silenced. Left: the number of spheres under each treatment. Right: representative images. Bar: upper 100 μm. Data were shown as mean + SD. ***P < 0.001.

We showed above that inhibition or depletion of p97 significantly reduced the expression of OCT4 and SOX2 in mammospheres (Figs. 2g, h and 3a). Indeed, treatments with Eer I and NMS-873 reduced *SOX2* levels in a time- and dose-dependent manner (Fig. 4a). *c-MYC* is a well-established oncogene and promotes pluripotency in both normal stem cells and CSCs^26^. Accordingly, we found that c-MYC expression is higher in MDA-MB-231 cells, as compared to MCF10A and MCF-7 cells (Fig. S5a). Inhibition and silencing of p97 with siRNA or dox-inducible *p97* shRNA greatly reduced c-MYC levels in MDA-MB-231 cells (Figs. 4b, c and S4b). In human breast cancer tissues, the level of c-MYC is positively correlated with that of p97 (Fig. 4d and Table S3). The reduction in c-MYC expression in p97-depleted cells is in agreement with our previous report that ER stress such as tunicamycin treatment decreases c-MYC expression^16^. c-MYC is known to activate the transcription of SOX2^27^. Reduced c-MYC expression in p97-depleted cells could further lead to decrease of SOX2.

*SKP2* is another important oncogene and CSC regulator, as high SKP2 expression predicts poor prognosis in multiple cancers including breast cancer^28^. Furthermore, deficiency or inhibition of SKP2 greatly restricts the CSCs of prostate cancer^29,30^. In MDA-MB-231 cells, inhibition or silencing of p97 with siRNAs or dox-inducible *p97* shRNA reduces SKP2 at both protein and mRNA levels (Figs. 4e, f, S4b, and S5b–d). In human breast cancer tissues, we also found a positive correlation between the levels of SKP2 and p97 (Fig. 4g and Table S4). Consequently, the levels of SKP2 also positively correlated with those of SOX2 (Fig. S5e). Like c-MYC, SKP2 expression is also downregulated upon ER stress^31^. Compared with adherent MDA-MB-231 cells, SKP2 expression was significantly higher in mammospheres (Figs. 4h and S5f).

Functionally, silencing of *SKP2* reduced SOX2 expression, the mammosphere formation and invasion of MDA-MB-231 cells (Figs. 4i, j and S5g). Interestingly, depletion of SKP2 little affected the p97 protein level, suggesting that SKP2 is a downstream target of p97 not vice versa (Fig. S5h). c-MYC is known as a transcription activator for SKP2^32^. Therefore in p97-depleted cells, reduced c-MYC could downregulate SKP2 expression. Together, these results suggest that loss of p97 also triggers the downregulation of other CSC regulators such as MYC and SKP2, in addition to SOX2 and OCT4. Importantly, the downregulation of SOX2, MYC, and SKP2 is interdependent.

### Loss of p97 activates UPR

**Fig. 5:**
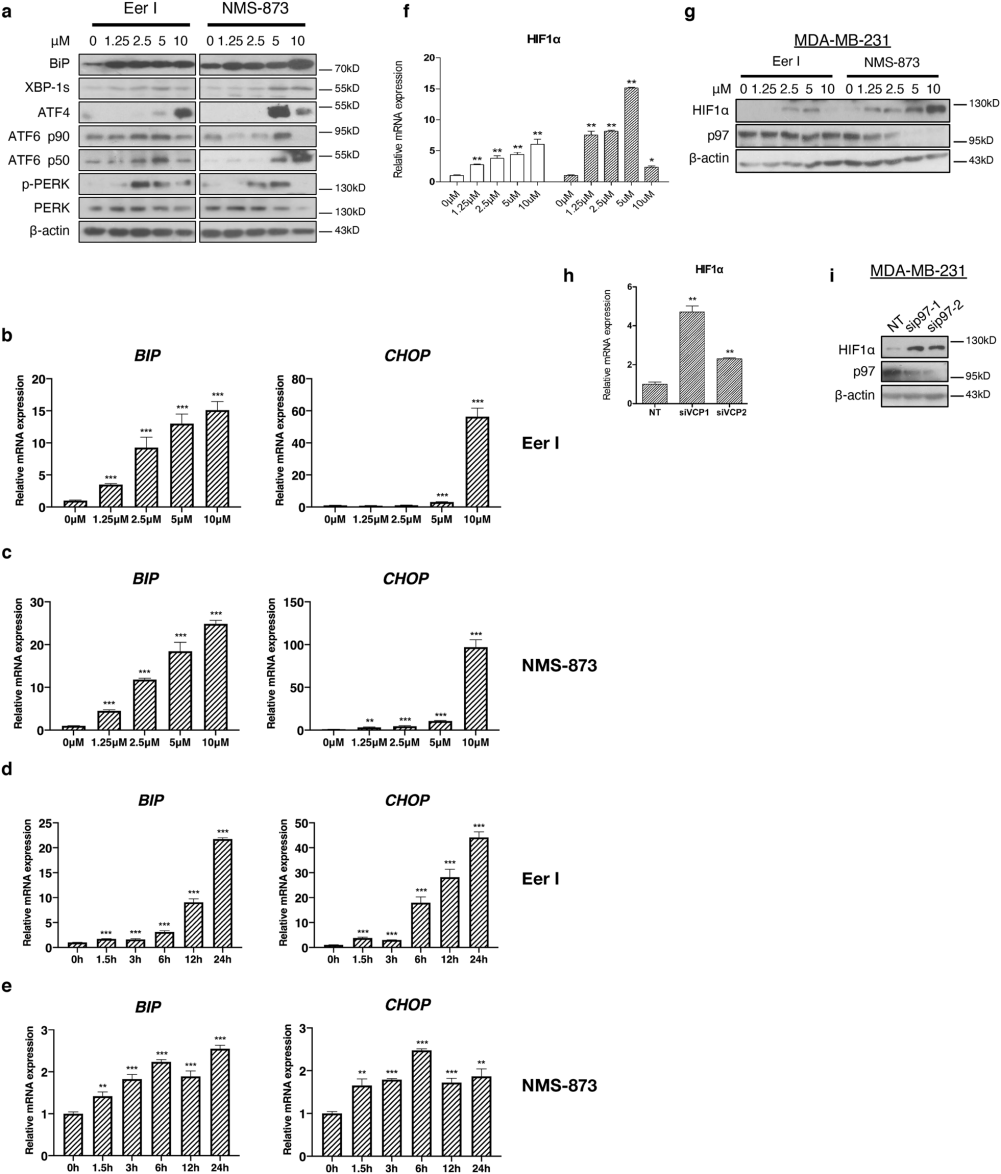
Loss of p97 activates UPR and increases HIF-1α expression. **a** Immunoblotting analysis of BIP, XBP1s, ATF4, ATF6 p90, ATF6 p50, PERK, and pPERK from the MDA-MB-231 cells treated with increasing concentrations of Eer I and NMS-873. β-actin serves as a loading control. **b**, **c** qPCR analysis of *BIP* and *CHOP* from the MDA-MB-231 cells treated with increasing concentrations of Eer I or NMS-873. **d**, **e** qPCR analysis of *BIP* and *CHOP* from the MDA-MB-231 cells treated with 2.5 μM Eer I or NMS-873 from 1.5 to 24 h. **f**, **g** qPCR and immunoblotting analysis of HIF-1α from the MDA-MB-231 cells treated with 2.5 μM Eer I or NMS-873. **h, i** qPCR and immunoblotting analysis of HIF-1α from the MDA-MB-231 cells with *p97* depleted. β-actin serves as a loading control. Data were shown as mean + SD. *P < 0.05, **P < 0.01, and ***P < 0.001.

Depletion or inhibition of p97 is known to induce ER stress^7,9,11^. Indeed, treatments with Eer I and NMS-873 greatly increased the levels of BIP (a major ER chaperon), spliced XBP1 (the active form), ATF4 and ATF6α (p90 and especially p50, the active fragment cleaved from p90), as well as PERK phosphorylation, in MDA-MB-231 cells (Fig. 5a). At mRNA levels, *BIP* and *CHOP* (a transcription factor for UPR and integrated stress response) were also strongly upregulated in a dose- and time-dependent manner after p97 inhibition (Fig. 5b–e). Together, these results demonstrate that treatment with Eer I and NMS-873 comparably activated the three branches of UPR. Hypoxia, a condition common to many solid tumors, closely interacts with ER proteostasis^33^. Hypoxia-inducible factor 1 (HIF-1α) is known to modulate UPR through interaction with XBP1 in triple negative breast cancers^34^. The mRNA and protein levels of HIF-1α were markedly increased in Eer I- and NMS-873-treated MDA-MB-231 cells as well as MDA-MB-231 cells with *p97* depleted (Fig. 5f–i). Similarly, HIF-1α expression was increased in MDA-MB-231 cells treated with tunicamycin, a glycosylation inhibitor and ER stress inducer (Fig. S5i).

### C/EBPδ is induced by loss of p97 and mediates the downregulation of stemness factors

**Fig. 6:**
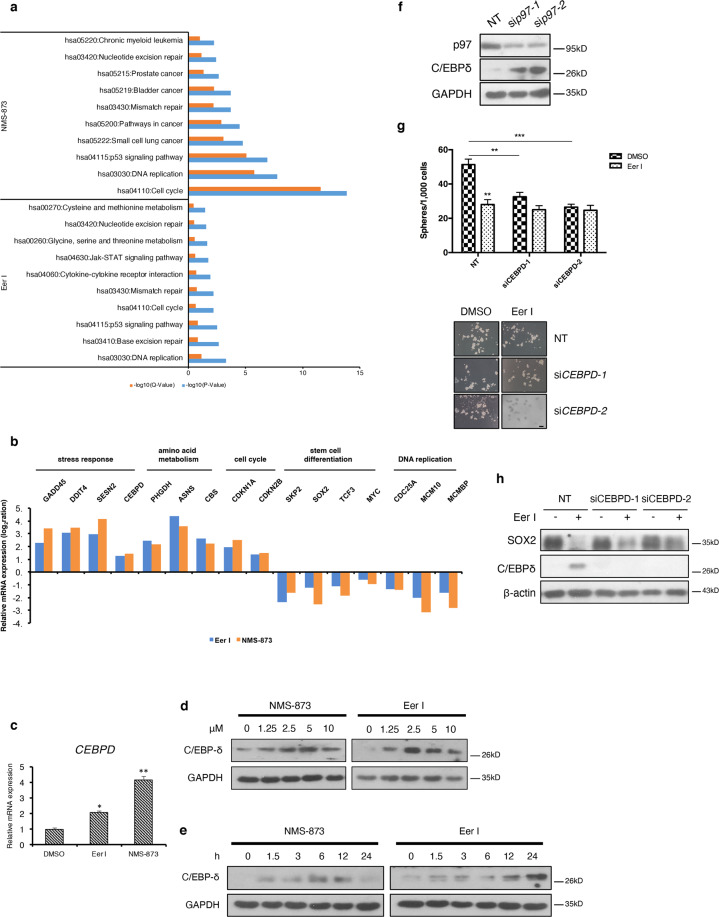
Loss of p97 upregulates C/EBPδ expression. **a** Pathways affected by p97 inhibition in MDA-MB-231 cells. **b** Microarray analysis of the representative up and downregulated genes. **c** qPCR analysis of *CEBPD* mRNAs from the MDA-MB-231 cells treated with 2.5 μM Eer I or NMS-873. **d**, **e** Immunoblotting analysis of C/EBPδ from the MDA-MB-231 cells treated with increasing concentration of Eer I or NMS-873 and the MDA-MB-231 cells treated with 2.5 μM Eer I or NMS-873 from 1.5 to 24 h. GAPDH serves as a loading control. **f** Immunoblotting of C/EBPδ from the MDA-MB-231 cells with *p97* silenced. GAPDH serves as a loading control. **g** Mammosphere formation of Eer I-treated MDA-MB-231 cells and with *CEBPD* silenced. Upper: the number of spheres under each treatment. Lower: representative images. Bar: 100 μm. **h** Immunoblotting of C/EBPδ and SOX2 from the Eer I-treated MDA-MB-231 cells with *CEBPD* silenced. Data were shown as mean + SD. *P < 0.05, **P < 0.01, and ***P < 0.001.

To better comprehend the cellular targets regulated by p97, we further examined the transcriptomic profiles of MDA-MB-231 cells treated with Eer I and NMS-873 using microarray. The DEGs from the inhibitor treatment group fell into several categories, including amino acid metabolism, stress response, DNA repair and recombination, and cancer (Fig. 6a). DEGs such as *SOX2*, *MYC*, *SKP2*, and *CEBPD* encode proteins known to influence the integrity and function of normal stem cells and CSCs (Fig. 6b). The CCAAT/enhancer binding protein delta (*CEBPD*, C/EBPδ) is a transcription factor involved in the modulation of multiple stress responses^35,36^. We previously showed that C/EBPδ is upregulated after UPR activation is inhibited and C/EBPδ is likely involved in the modulation of CSCs^16^. Similarly, the mRNA and protein levels of *CEBPD* were markedly increased in Eer I- and NMS-873-treated MDA-MB-231 cells (Fig. 6c–e). Silencing of *p97* also increased C/EBPδ levels in MDA-MB-231 cells (Fig. 6f). These findings suggest an inverse correlation between p97 and C/EBPδ. Functionally, silencing of *CEBPD* reversed the inhibitory effects of Eer I on mammosphere formation (Fig. 6g), as well as partly reversing the decrease in SOX2 expression (Fig. 6h). These results suggest that C/EBPδ could act downstream of p97 in the CSC control.

Among the several known mechanisms upregulating *CEBPD* expression, HIF-1α is a potent activator^36,37^. The elevation of HIF-1α after UPR activation explains at least in part the induction of C/EBPδ in p97-inhibited cancer cells. The elevated C/EBPδ level in turn likely contributes to the decrease of c-MYC and SKP2 expression in p97-depleted cells, as several studies demonstrated that C/EBPδ downregulates the expression of c-MYC and SKP2^36,38^. Thus, the increase in C/EBPδ triggered by loss of p97 likely contributes to the demise of the CSCs in breast cancer.

## Discussion

In this study, we first showed that, compared with the non-CSC cancer cells, p97 expression is significantly higher in the CSC population in both human breast cancer tissues and breast cancer cells cultured in vitro. Consistent with proposed importance in tumorigenesis, p97 levels also positively correlate with the histological grade, tumor size, and lymph node metastasis in human breast cancer samples. We then showed that p97 is critical for tumor growth, invasion, the integrity of CSC population, and CSC-enriched mammospheres. The CSC population is significantly more sensitive to the loss of p97, suggesting that p97-mediated proteostasis control and ERAD play a greater role in these cells. Mechanistically, loss of p97 exacerbates ER stress and activates UPR, which in turn induces C/EBPδ expression and downregulates c-MYC, SOX2, and SKP2.

There is already a host of evidence demonstrating that proteostasis control system, such as the ubiquitination enzymes, proteasome, and chaperones, is crucial for embryonic and adult stem cells to maintain pluripotency^18,39,40^. Cdc48, the evolutionary ortholog of p97, is indispensable in many developmental processes in *S. cerevisiae*, *C. elegans*, and *Drosophila*^17^. Homozygous p97^−/−^ mice died at a periimplantation stage, indicating that proteostasis control by p97 is essential for early embryo development^41^. Our findings from this study demonstrate that p97-mediated proteostasis control and ERAD are essential for the CSCs. In line with our findings, silencing of p97 or treatment with NMS-873, DBeQ, and Eer I aggravated ER stress and selectively killed the HER2-positive breast cancer cells^42^.

The fact that blocking ERAD alone is sufficient to impair cancer stemness highlights the significance of ER proteostasis to the CSCs and cancer development. We previously showed that UPR activation is elevated in the breast CSC population^16^. In a recent report, Dillin and colleagues also showed that transient activation of UPR is necessary for the reprogramed somatic cells to acquire pluripotency^43^. Prolonged ER stress, which triggers apoptosis, gravely impairs the breast CSC population^16^. Because p97 is indispensable in ERAD, loss of p97 eventually leads to the pro-apoptopic response and cell death in cancer cells. C/EBPδ influences the differentiation of normal stem cells and CSCs^44,45^. C/EBPδ binds to the promoters of *SOX2*, *OCT4*, *MYC,* and other genes involved in stemness, thereby affecting pluripotency and the CSC population^45^. Interestingly, many stemness and pluripotency factors seem to reciprocally regulate each other. For example, increase of C/EBPδ downregulates c-MYC, while MYC and the MYC-interacting protein MIZ1 repress C/EBPδ expression^46^. Such a regulatory loop also exists for C/EBPδ and HIF-1α^37^. We propose that the steady increase of C/EBPδ and precipitous decrease of c-MYC in p97-inhibited or depleted cells lead to reduced expression of SOX2 and SKP2, which underscores the drastic demise of the breast CSC population.

Because p97 interacts with a myriad of proteins, additional CSC-modulating targets likely exist, which was also implicated by our microarray study (Fig. 6a, b). For example, intracellular amino acid levels are critical for stem cell differentiation^47^. Recently, several studies reported that p97 plays an important role in amino acid homeostasis. Treatment with NMS-873 and DBeQ altered the levels of multiple amino acids in lung carcinoma A549 cells^48^. In addition, p97 promotes the degradation of glutamine synthetase^49^ and coordinates with GCN2, an amino acid-sensing kinase, to maintain metabolic homeostasis and proteostasis in cancer cells^50^.

## Conclusions

Our findings show that p97 regulates the CSC population through multiple mechanisms, many of which are induced by UPR activation. This study illustrates another layer of p97 regulation in cancer development, which has not been previously defined, and reaffirms the strategy of p97 antagonism in cancer treatment.

## Supplementary information

Table S1-S5

Supplementary Figure Legends

Figure S1

Figure S2

Figure S3

Figure S4

Figure S5

## Data Availability

The experimental material and data from this study will be available upon request.
