# Supplementary material for: p97/VCP is highly expressed in the stem-like cells of breast cancer and controls cancer stemness partly through the unfolded protein response
Source: Cell Death Dis. 2021 Mar 17;12(4):286. doi: 10.1038/s41419-021-03555-5 (PMC7969628; doi:10.1038/s41419-021-03555-5)
Supplement: Supplementary file 1 — Table S1-S5 [file 41419_2021_3555_MOESM1_ESM.pdf]

Supplementary Tables

Table S1. Correlation between the levels of p97 and $CD44^{+}/CD24^{-}$ CSC population in breast cancer patients (n = 75).

	Expression	$CD44^{+}/CD24^{-}$	non $CD44^{+}/CD24^{--}$	$CD44^{-}/CD24^{-}$	$CD44^{-}/CD24^{+}$	$CD44^{+}/CD24^{+}$	*p*-value
p97	High	22	17	17	0	0	< 0.001
	Low	10	61	58	2	1	

p-values were calculated using the chi-squared test.

Table S2. Correlation between the levels of p97 and SOX2 in breast cancer patients (n = 75).

	SOX2			
	Expression	Low	High	*p*-value
p97	Low	21	20	< 0.001
	High	3	31	

p-values were calculated using the chi-squared test.

Table S3. Correlation between the levels of p97 and c-MYC in breast cancer patients (n = 98).

	c-MYC			
	Expression	Low	High	*p*-value
p97	Low	32	9	< 0.001
	High	4	53	

p-values were calculated using the chi-squared test.

Table S4. Correlation between the levels of p97 and SKP2 in breast cancer patients (n = 97).

	SKP2			
	Expression	Low	High	*p*-value
p97	Low	37	4	< 0.001
	High	3	53	

p-values were calculated using the chi-squared test.
