# Supplementary material for: p97/VCP is highly expressed in the stem-like cells of breast cancer and controls cancer stemness partly through the unfolded protein response
Source: Cell Death Dis. 2021 Mar 17;12(4):286. doi: 10.1038/s41419-021-03555-5 (PMC7969628; doi:10.1038/s41419-021-03555-5)
Supplement: Supplementary file 2 — Supplementary Figure Legends [file 41419_2021_3555_MOESM2_ESM.pdf]

Supplementary Figure Legends

Figure S1 P97 inhibition reduced the growth of HepG2, HCT-116 and PANC-1 cells.

HepG2, HCT-116 and PANC-1 cells were treated with 1.25, 2.5, 5 and 10 μM Eer I or NMS-873 for 24 hr and cell viability was measured. IC50 values were indicated. DMSO was used as a vehicle control.

Figure S2 p97 inhibition reduces breast cancer cell invasion and orthotopic growth in nude mice

a Eer I reduced MDA-MB-231 cell invasion. Left: representative images of crystal purple staining. Right: percentage of invaded cancer cells relative to the DMSO-treated cells. Bar: 100 μm. Data were shown as mean + SD. **b** Representative staining of PCNA, cleaved CASPASE-3, CD31, CD44, CD24 and OCT4 in control or Eer I-treated tumors resected from nude mice. Bar: 100 μm. **c** Upper: Flow cytometry analysis of CD44 and CD24 CSC population in control or Eer I-treated tumors resected from nude mice. Lower: Percentage of $CD44^{+}/CD24^{-}$ CSC population. Data were shown as mean + SD. **P < 0.01, ***P < 0.001.

Figure S3 $ALDH^{+}$ CSCs are much more sensitive to p97 inhibition than non-CSC cells in vivo.

a $ALDH^{+/high}$ and $ALDH^{-/low}$ cells isolated from MDA-MB-231 cells were inoculated into nude mice. Tumors treated with DMSO, Eer I or NMS-873 at the time of sacrifice, day 56. **b** Weight of these tumors Data were shown as mean + SD. **P < 0.01, ***P < 0.001.

Figure S4 Doxycycline (dox)-induced silencing of *p97* reduces mammosphere formation and MYC and SKP2 expression.

MDA-MB-231 cells were transfected with a Dox-inducible construct encoding *p97* shRNA. **a** Representative images of spheres treated in presence or absence of Dox for 7 days. **b**

Immunoblotting analysis of p97, c-MYC from MDA-MB-231 cells were transfected with Dox as indicated. Bar: 100μm.

Figure S5 Expression of p97 c-MYC, SOX2, SKP2 and HIF-1α in breast cancer cells

a Immunoblotting analysis of c-MYC and p97 from MCF10A, MCF-7 and MDA-MB-231 cells. β-actin serves as a loading control. **b** qPCR analysis of *SKP2* from MDA-MB-231 cells treated with increasing concentration of Eer I and NMS-873. **c** qPCR analysis of *SKP2* from MDA-MB-231 cells treated with 2.5 μM Eer I and NMS-873 from 1.5 to 24 hrs. **d** Immunoblotting analysis of SKP2 from MDA-MB-231 cells treated with 2.5 μM Eer I and NMS-873 from 1.5 to 24 hrs. β-actin serves as a loading control. **e** SKP2 expression correlates with that of SOX2 in human breast cancer tissues. Left: representative immunohistochemical staining of SKP2 and SOX2 in the consecutive serial sections of breast cancer tissues. SKP2 and SOX2 expressions were classified as low or high. Bar: upper 200 μm, lower 50 μm. Right: correlation analyses of SKP2 and SOX2 expression in breast cancer tissues (n = 75). **f** qPCR analysis of *SKP2* from the adherent and spheroid MDA-MB-231 cells. **g** Depletion of *SKP2* reduced MDA-MB-231 cell invasion. Left: representative image of crystal purple staining. Bar: 100μm. Right: percentage of invaded cancer cells. **h** Immunoblotting analysis of p97 and SKP2 from MDA-MB-231 cells with *SKP2* silenced. β-actin serves as a loading control. **i** Immunoblotting analysis of HIF-1α in the MDA-MB-231 cells treated with increasing concentrations of tunicamycin. β-actin serves as a loading control. Data are shown as mean + SD. *P < 0.05, **P < 0.01, ***P < 0.001.
