# Supplementary figures and images for: p97/VCP is highly expressed in the stem-like cells of breast cancer and controls cancer stemness partly through the unfolded protein response
Source: Cell Death Dis. 2021 Mar 17;12(4):286. doi: 10.1038/s41419-021-03555-5 (PMC7969628; doi:10.1038/s41419-021-03555-5)

Fig. S1

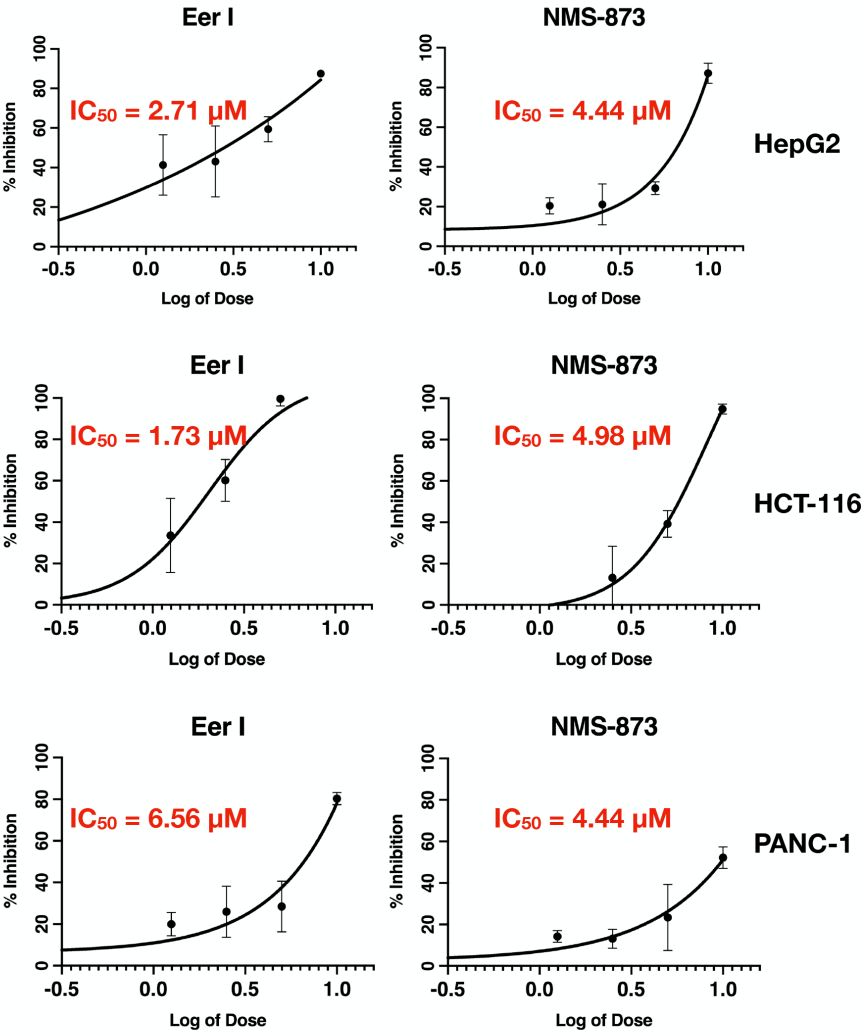

Supplement: Supplementary file 3 — Figure S1 [file 41419_2021_3555_MOESM3_ESM.pdf]

Fig. S2

a

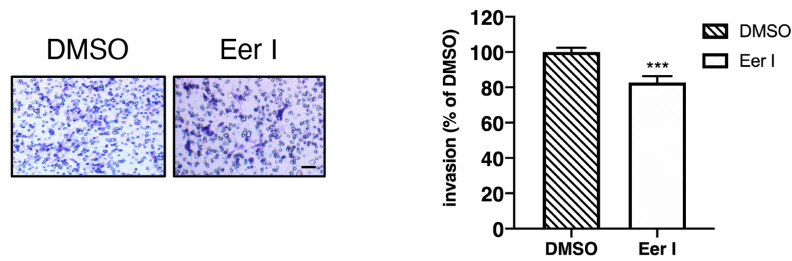

b

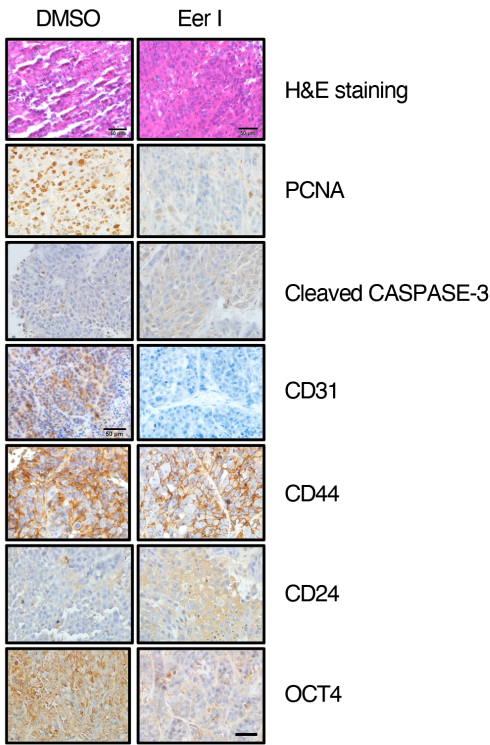

c

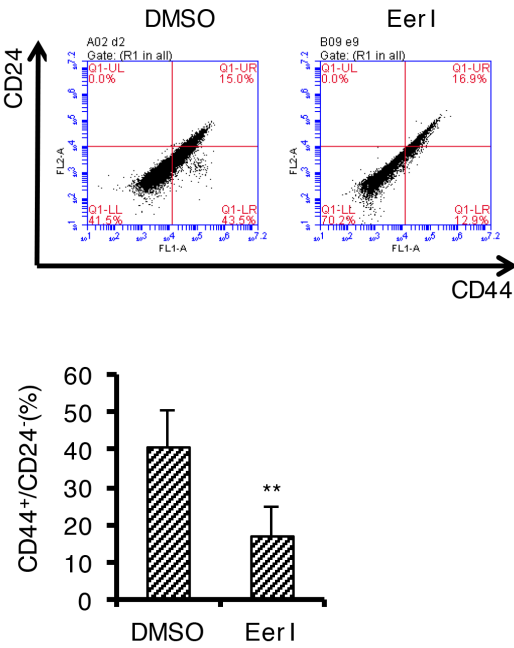

Supplement: Supplementary file 4 — Figure S2 [file 41419_2021_3555_MOESM4_ESM.pdf]

Fig. S3

a

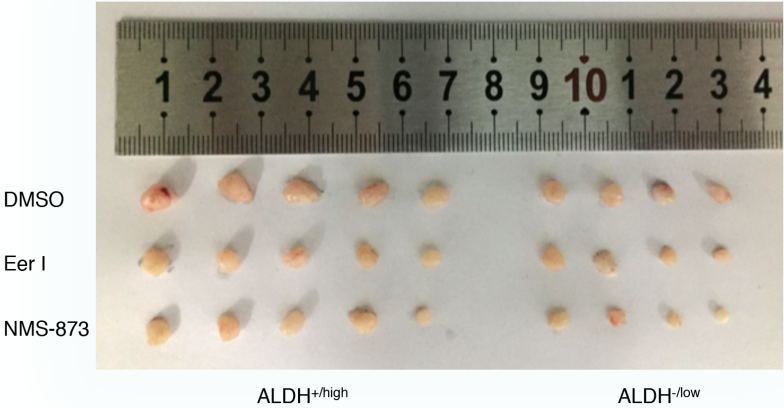

b

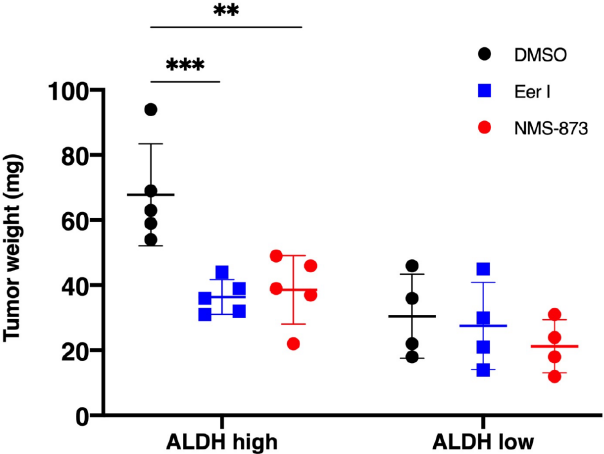

Supplement: Supplementary file 5 — Figure S3 [file 41419_2021_3555_MOESM5_ESM.pdf]

Fig. S4

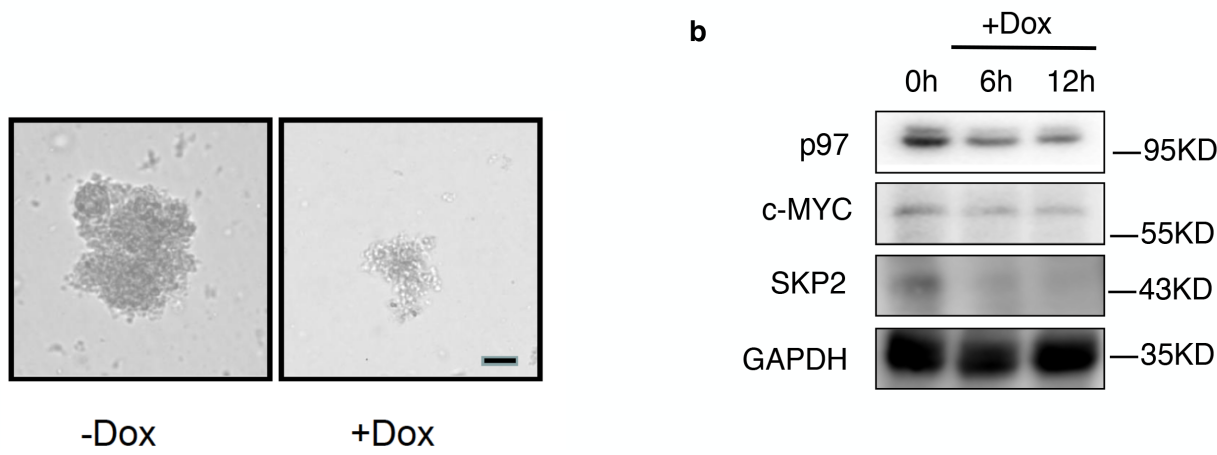

Supplement: Supplementary file 6 — Figure S4 [file 41419_2021_3555_MOESM6_ESM.pdf]

Fig. S5

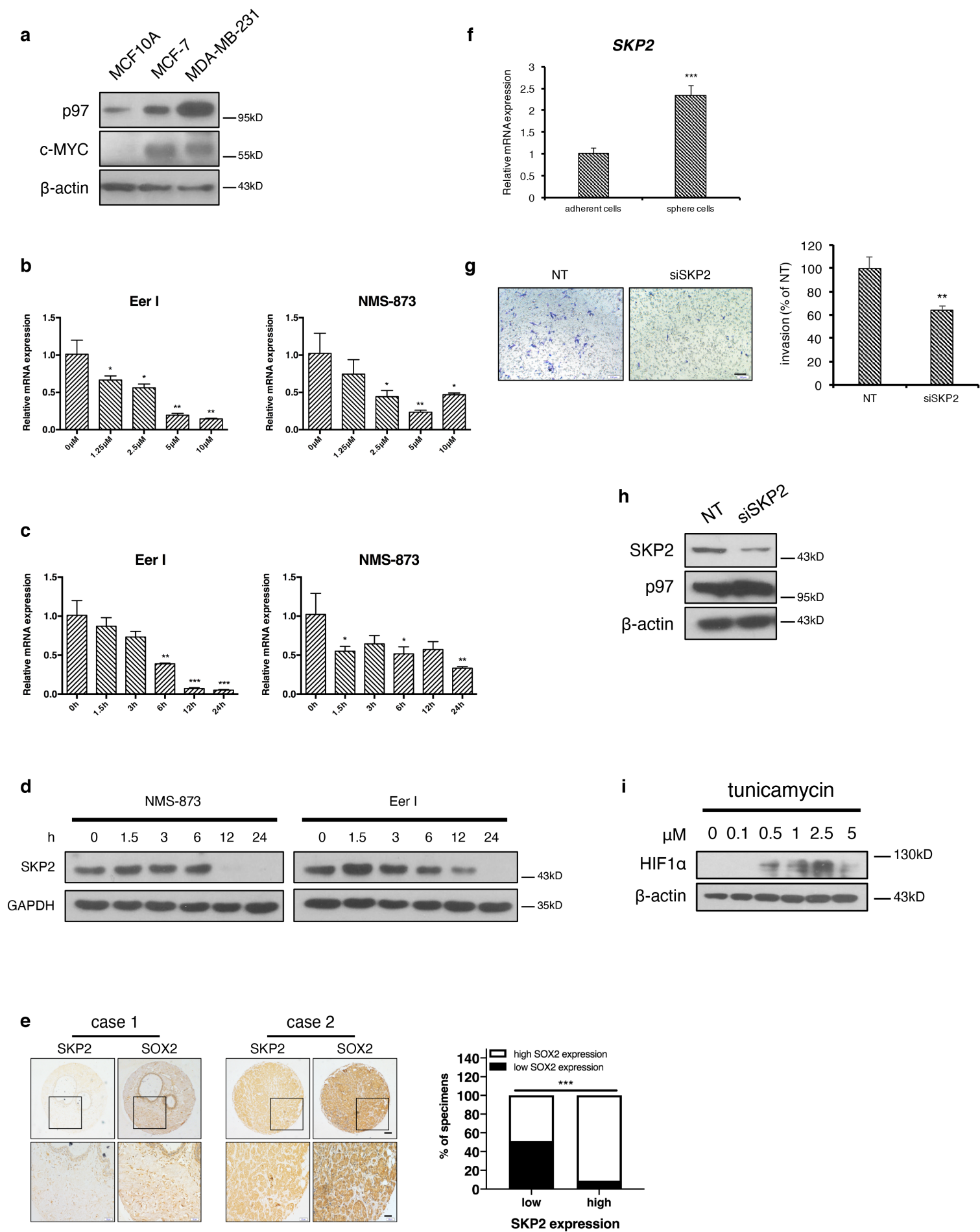

Supplement: Supplementary file 7 — Figure S5 [file 41419_2021_3555_MOESM7_ESM.pdf]
